# Clinical Features and Outcomes of Coronavirus Disease 2019 in Patients with Inflammatory Bowel Disease and Spondyloarthropathies

**DOI:** 10.5152/tjg.2022.22181

**Published:** 2022-09-01

**Authors:** Mukaddes Tozlu, Gamze Dilek, Mehtap Kalçık Unan, Ayhan Kamanlı, İbrahim Tekeoğlu, Mustafa İhsan Uslan, Kemal Nas

**Affiliations:** 1 Department of Gastroenterology, Sakarya University Faculty of Medicine, Sakarya, Turkey; 2 Division of Rheumatology, Department of Physical Medicine and Rehabilitation, Sakarya University Faculty of Medicine, Sakarya, Turkey

**Keywords:** Coronavirus disease 2019, inflammatory bowel disease, psoriatic arthritis, rheumatic diseases

## Abstract

**Background:**

We aimed to determine the clinical features, predictive factors associated with severe disease, and outcomes of coronavirus disease 2019 in patients with immune-mediated inflammatory diseases and report data on the comparison of coronavirus disease 2019 between patients with inflammatory bowel disease and spondyloarthropathies.

**Methods:**

A total of 101 patients with inflammatory bowel disease and spondyloarthropathies who had confirmed diagnosis of coronavirus disease 2019 were retrospectively analyzed. Demographics, comorbidities, immunosuppressive treatments, and the impact of immunosuppression on negative outcomes were assessed.

**Results:**

The median age of the patients was 47 (38-57) years. The most common rheumatologic diagnosis was ankylosing spondylitis (n = 24), psoriatic arthritis (n = 17), and reactive arthritis (n = 1). In the inflammatory bowel disease group, 47 patients had ulcerative colitis, 11 Crohn’s disease, and 1 unclassified. The most commonly used treatments were biologics (55%) in the spondyloarthropathies group and aminosalicylates (66.1%) in the inflammatory bowel disease group. Overall, 18.8% of the patients required hospitalization, 5% developed severe complications, and 2% died. There were no significant differences in coronavirus disease 2019-related negative outcomes between spondyloarthropathies and inflammatory bowel disease patients. The median age was higher in the patients who required hospitalization [57 (46-66) vs 47 (38-57) years, *P* = .008]. Bilateral opacities on chest radiographs were more common in the patients who required hospitalization in the spondyloarthropathies group [88.9% vs 14.3%, *P* = .016]. Comorbidity was significantly associated with hospitalization in the inflammatory bowel disease group (*P* ≤ .05). Baseline therapy with biologics or immunosuppressives was not associated with severe coronavirus disease 2019 outcomes.

**Conclusion:**

Older age, comorbidities, and bilateral ground-glass opacities were associated with adverse outcomes, whereas specific immune-mediated inflammatory disease diagnoses or immunosuppressive treatments were not.

**Main Points**

- Patients with immune-mediated inflammatory diseases (IMIDs) are the delicate population during the pandemic because of the immunosuppressive condition and treatments.
- Specific IMIDs diagnoses or immunosuppressive treatments were not associated with adverse coronavirus disease 2019 (COVID-19) outcomes. Biologics were not found to have a protective effect on adverse outcomes related to COVID-19.
- There were no significant differences in COVID-19-related negative outcomes between patients with spondyloarthropathy and inflammatory bowel disease.

## Introduction

Coronavirus disease 2019 (COVID-19), caused by the coronavirus severe acute respiratory syndrome coronavirus-2 (SARS-CoV-2), is an ongoing health crisis worldwide. The clinical manifestations and outcomes range from asymptomatic or benign courses to acute respiratory distress syndrome (ARDS), multi-organ failure, and finally death.^1^ The risk of severe disease and mortality is higher in elderly patients and those with underlying comorbidities.^2^

Inflammatory bowel diseases (IBD), spondyloarthropathies (SpA), and related rheumatologic autoinflammatory conditions are chronic, immune-mediated inflammatory diseases (IMIDs) affecting millions of people worldwide. These patients are expected to be more susceptible to COVID-19 or more severe clinical course because of their increased infectious risk, dysregulated immune response, and immunosuppressive or immunomodulatory therapies.^3^ There are particular concerns regarding the risk of infection and consequences of COVID-19 in patients with IMIDs. Some immunosuppressive agents such as prednisolone and methotrexate were related to higher hospitalization risk among patients with COVID-19 and IMIDs^4,5^

So far, the impact of COVID-19 in patients with IMIDs has not been fully understood. There are limited data about which immunosuppressive therapies or factors may increase the susceptibility to infection and may predict poor outcomes.

In this study, we aimed to evaluate the impact of comorbidities and medications on COVID-19 outcomes in patients with IMIDs. This study was conducted in our gastroenterology and rheumatology departments and provided a detailed observational report comparing clinical characteristics, risk factors, and outcomes of COVID-19 in patients with SpA and IBD.

## Materials and Methods

This was a single-center, retrospective cohort study evaluating the clinical features, disease course, and outcomes of laboratory-confirmed COVID-19 cases among patients with IMIDs including SpA and IBD, followed at an academic gastroenterology and rheumatology departments in our tertiary center. The study was conducted between March 2020 and August 2021.

We used 2 study populations to identify the clinical characteristics of COVID-19. First, we evaluated all adult patients in our gastroenterology department with the diagnosis of IBD [including ulcerative colitis (UC) and Crohn’s disease (CD)] for at least 6 months and who had a confirmed diagnosis of COVID-19. The second study population came from a large SpA patient cohort followed in our rheumatology department. A confirmed diagnosis of COVID-19 was defined as a positive SARS-CoV-2 polymerase chain reaction (PCR) test in a nasopharyngeal swab. Patients were identified by searching for COVID-19 and IBD, and SpA diagnosis codes, as well as by manually screening all patients in our updated IBD and SpA patients database. Laboratory and radiological findings were obtained from electronic medical records. Clinical data from patients were obtained during hospital visits face-to-face or via direct telephone call by the principal investigator.

We collected the following information for all eligible patients: patient demographics, comorbidities, IBD and SpA characteristics (disease type, activity at the time of COVID-19 diagnosis, treatment, adherence to the ongoing treatment or discontinuation, and changes in disease activity), signs and symptoms related to COVID-19, laboratory data, therapies for COVID-19, and outcomes (need for hospitalization, admission to intensive care units, length of hospital stay, oxygen therapy, the requirement of respiratory support, complications, and mortality). Clinical activity of IBD was evaluated with Harvey–Bradshaw Index and Mayo score for CD and UC, respectively.^6,7^ Laboratory data included the following variables: complete blood cell (including lymphocyte counts), liver function tests, renal function tests, C-reactive protein (CRP), albumin, D-dimer, ferritin, and interleukin-6 (IL-6).

The primary objective was to investigate the features of COVID-19 in patients with SpA and IBD in terms of signs and symptoms, hospitalization, therapy, and death.

This study was conducted in accordance with the principles of the Declaration of Helsinki and the ethical standards of the responsible committee. The study was approved by the ethics committee of Sakarya University Faculty of Medicine (August 27, 2021, no: 444). Informed consent was obtained from all participants.

### Statistical Analysis

Descriptive analyses were presented using medians and interquartile range (IQR) for the non-normally distributed variables. Student’s *t*-test and the Mann–Whitney *U*-test were used to analyzing normally distributed continuous data and non-normally distributed data, respectively. Chi-square test was used to compare the categorical variables and risk analyses between two groups. The categorical variables were reported as frequency (%). *P* < .05 was considered significant. Statistical Packages for Social Sciences version 21 (IBM Corp.; Armonk, NY, USA) was used for the analyses.

## Results

### Study Population

We analyzed the clinical features of 101 patients with SpA and IBD who had confirmed diagnosis of COVID-19. The diagnosis of IBD patients was UC in 47 patients (46.5%), CD in 11 (10.9%), and unclassified in 1. The diagnosis of patients with rheumatic diseases was ankylosing spondylitis (AS) in 24 patients (23.8%), psoriatic arthritis (PsA) in 17 (16.8%), and reactive arthritis in 1. The median age of the IBD and SpA patients was 46 (36-57) and 48 (40-57) years, respectively. In the overall population, 42.5% of the patients had underlying comorbidity. Comorbidity was equally distributed among SpA and IBD patients. Three patients had concomitant immune-mediated diseases in the IBD group (2 psoriases and 1 rheumatoid arthritis).

Regarding ongoing treatments for IBD, most patients were on aminosalicylates (66.1%) and conventional synthetic disease-modifying antirheumatic drugs (csDMARDs) (13.5%), followed by biologic agents (alone or combo) (10.2%) and low-dose corticosteroids (CS) (3.4%) before the onset of COVID-19 symptoms. Regarding therapy previously used in SpA group, most patients were on biologics (55%), followed by csDMARDs (23.8%) and low-dose CS (7.2%). The demographics and clinical characteristics of the included patients are shown in Table 1.

On admission, leucopenia or lymphopenia was not detected in laboratory tests. There was a moderate elevation in D-dimer, ferritin, and CRP values. There was no significant difference between IBD and SpA groups in laboratory findings except for alanine aminotransferase level. Alanine aminotransferase level was higher in patients with SpA than in patients with IBD (*P* = .013). Laboratory findings at the time of COVID-19 diagnosis are shown in Table 2.

### Manifestations of Coronavirus Disease 2019 Infection

Concerning the COVID-19 symptoms, the most common symptom was arthralgia/myalgia (73%). Dysosmia and dysgeusia were seen in 40% and 42% of the patients, respectively. There was no difference between SpA and IBD patients with regard to the symptoms except for the fever. Gastrointestinal symptoms were not frequent. Diarrhea occurred in 19% of the patients.

After recovery from COVID-19, 17 (16.8%) patients reported persistent symptoms which include joint pain (35.2%), dyspnea (17.6%), fatigue (11.7%), vertigo (5.8%), chest pain (5.8%), cough (11.7%), anosmia (5.8%), dysgeusia (5.8%), and forgetfulness (5.8%).

### Consequences of Coronavirus Disease 2019 on Immune-Mediated Inflammatory Diseases

The median interval between symptoms onset and PCR confirmation was 2 days (IQR: 1.5-3.5). Bilateral abnormalities on chest radiography or computed tomography were found in 20.8% of the patients. Twenty-three (22.8%) patients had radiograph findings compatible with pneumonia.

Overall, 82 (81.2%) patients with mild disease were isolated at home, and 19 (18.8%) were hospitalized. There was no difference between the SpA and IBD groups regarding hospitalization (*P* = .278). Out of 19 patients who required hospitalization, 5 (26%) patients developed severe complications (4 ARDS and 1 acute kidney injury), 3 (15.7%) required mechanical ventilation, and 15 (78.9%) required low-flow oxygen supplementation. A total of 2 patients died of COVID-19. The clinical results of the patients by comparison between groups are shown in Table 3.

Among patients hospitalized in the SpA group, 50% were under treatment with csDMARD, 10% low-dose CS, 40% antitumor necrosis factor (TNF)-alpha (1 anti-TNF only and 3 combined with csDMARD). In the IBD group, 77.8% of the patients were on 5-aminosalicyclic acid treatment (5-ASA) and 22.2% on thiopurines. Two patients with SpA were under treatment with JAK inhibitör and did not require hospitalization. No significant differences were observed in concomitant medications between patients with SpA and IBD who required hospitalization (*P* = .161).

In addition, 85% of the patients received treatment for COVID-19. Favipiravir (80.8%), antibiotics (16%), hydroxychloroquine (4%), low molecular weight heparin (LMWH) (21%), dexamethasone (7.1%), and oseltamivir (1%) were prescribed either in monotherapy or in combination. Two patients (2%) had tocilizumab, and 1 had convalescent plasma therapy. Fifteen (15.5%) patients received no treatment.

### Factors Associated with Severe Course

On analyzing the characteristics of patients who required hospitalization, there were no statistically significant differences between the 2 groups (hospitalized vs. non-hospitalized) in gender, smoking, rheumatologic diagnosis, disease duration, and baseline treatment (Table 3). Overall, the median age was higher in the patients who required hospitalization than the patients who did not [57 (46-66) vs 47 (38-57) years, respectively (*P* = .008)]. Bilateral opacities on chest radiograph were more common in the patients who required hospitalization in the SpA group (*P* = .016). Among the hospitalized patients, 57.8% had at least 1 comorbidity; hypertension (36.8%) and diabetes mellitus (26.3%) were the most common. Comorbidity was significantly associated with hospitalization in IBD group (n = 7/9, 77.8%, *P* ≤ .05) and not in SpA group (n = 4/10, 40%, *P* = .199). Patients on biologics or immunosuppressives did not have more severe COVID-19 outcomes. Two patients who died were on mesalazine therapy. One of them received tocilizumab.

Overall, 47 (46.5%) patients (80.5% in the SpA group and 24.6% in the IBD group) suspended their medication due to COVID-19. Regarding anti-TNF therapy, 2 patients in the SpA group and 4 in the IBD group continued their medication and did not experience adverse outcomes. Of the 29 patients undergoing biologics, 23 (79.3%) postponed their infusion. No significant recurrence of rheumatic disease was observed in the patients who modified their treatment. Ten patients (17%) in the IBD group and 14 (33%) in the SpA group had active disease at the beginning of COVID-19. Three patients reported a flare of disease activity in the IBD group with COVID-19. One patient required initiation of systemic steroids.

### Vaccination Status

Of the included patients, 88.6% had no vaccination, 2.3% had 1 dose, 8% had 2 doses of inactivated SARS-CoV-2 vaccine, 1.1% had 1 dose of mRNA-based vaccine before COVID-19 diagnosis, 24.1% of the patients had no vaccines before or after COVID-19 diagnosis, 20.8% of the patients had inactivated vaccine, and 43.7% had mRNA-based vaccine after the COVID-19 diagnosis. We did not detect any recurrent COVID-19 infection in our cohort.

## Discussion

The pathogenesis and disease course of COVID-19 are still under investigation. Immune-mediated inflammatory diseases present a real challenge in the COVID-19 pandemic. One might think that intrinsic immune dysregulation in chronic inflammatory conditions and medications used in the treatment may increase the risk of infection and severe outcomes. However, data about the specific risk factors and disease course in patients with IMIDs are yet to be determined.

Spondyloarthritis and IBD are distinct chronic inflammatory conditions characterized by some degrees of overlap in genetic factors, clinical symptoms, and pathogenesis. Although both share the common pathogenic mechanisms, it was shown that patients with SpA and IBD differ in circulating IL-17/IL-23 axis cytokines and integrins, regardless of symptoms.^8^ In addition, these conditions are separate clinical entities; while SpA mainly damages the spine and/or peripheral joints, IBD primarily affects the gastrointestinal tract. Some therapeutic agents proven effective in CD treatment do not appear to work in AS, such as IL-12/ IL-23 monoclonal antibody.^9^ In this study, we aimed to report how patients with IMIDs from a tertiary care hospital were affected by COVID-19 and reveal similar or different aspects of these 2 diseases (SpA and IBD) in terms of COVID-19-related adverse outcomes by comparing the characteristics of these patients. To our knowledge, this study is the first to date to examine the characteristics of IBD patients compared to a cohort of patients with SpA who had a confirmed diagnosis of COVID-19. We have found similar hospitalization rates in the patients with IBD and SpA with COVID-19. This study also includes the second largest cohort of PsA patients with COVID-19 in the literature.

Some studies have shown that patients with IBD are not at increased risk of infection as the general population.^10,11^ However, conflicting clinical results were found about immunosuppressive therapies.^12-14^ Overall, whether immunosuppressive therapies enhance the risk or protect from the development of severe COVID-19 is still uncertain.

In most studies, including ours, comorbidities and older age have been found to be independent risk factors for COVID-19-related poor outcomes in patients with IMIDs.^13,14^ Moreover, we identified no association between baseline treatment, including immunosuppressive therapies, and hospitalization risk among SpA and IBD patients with COVID-19, similar to some studies.^15-17^ In a study conducted by Bakasis et al^18^, COVID-19 manifestations, outcomes, and antibody responses to SARS-CoV-2 were investigated among patients with autoinflammatory rheumatic diseases. Almost two-thirds of the patients had a mild course of the disease, 23.4% were hospitalized, and the mortality rate was 1.2%. The patients with a more severe course were older, had a medical history of lung interstitial pathology, and were on corticosteroids, rituximab, and mycophenolate mofetil. In an Italian study, therapy with biologics and immunosuppressants was not associated with worse outcomes, even though a trend toward a worse prognosis was reported with corticosteroid use.^19^ The COVID-19 Global Rheumatology Alliance physician-reported registry^14^ showed that glucocorticoids of ≥10 mg/day are associated with an increased risk of hospitalization. However, TNF antagonists decreased the risk of hospitalization in patients with rheumatic disease. SECURE IBD,^4^ the largest registry regarding IBD and COVID-19, reported that aminosalicylates and systemic corticosteroids were associated with a higher risk of hospitalization and severe outcomes, but anti-TNFs and other biologics were not independent risk factors for more severe COVID-19.

The development of a cytokine release syndrome with persistent viral stimulation leads to a significant release of cytokines such as IL-6 and TNFa, which can trigger end-organ damage during COVID-19 infection.^20^ It is suggested that the severe course in the patients infected with SARS-CoV-2 may be due to an uncontrolled immune response. This uncontrolled immune status may change the course of chronic inflammatory diseases. A case of psoriasis that flared with COVID-19 was reported by Ozaras et al.^21^ The authors suggested that hyperinflammation status generated by COVID-19 might exacerbate psoriasis. It is possible that immunomodulators could decrease the immune response by controlling the hyperinflammatory state and, consequently, lead to a better prognosis. This might explain why TNF antagonist treatment is not associated with adverse COVID-19 outcomes, as shown in some studies.^4^ It was suggested that anti-TNF monotherapy might have a protective effect against severe COVID-19 by blunting the cytokine storm related to severe disease.^22^ In fact, we found neither increased nor decreased risk of adverse outcomes with biologic use.

There is not much data on PsA patients with COVID-19 in the literature. A case of COVID-19 was described in a patient with psoriatic arthritis and CD treated with an IL-23 inhibitor. No change in the course of PsA was reported during the COVID-19 infection. The authors suggested that IL23/IL-17 axis inhibition might not be harmful in the case of SARS-COV-2 infection.^23^ In another case report, it was presented that a patient with PsA and hypertension treated with adalimumab rapidly recovered from COVID-19 without complications. No change in the course of the disease has been reported.^24^ Similarly, we did not observe the detrimental effect of biologics or any increased risk of disease activation due to COVID-19 in our PsA patients.

It was shown in most studies that vaccinated patients with systemic rheumatic diseases with COVID-19 have better outcomes compared with unvaccinated counterparts.^25,26^ Because our retrospective analysis included all patients before vaccination started, most of the patients had no vaccination before COVID-19 infection.^27^ Two-thirds of the patients had vaccination after the COVID-19 infection. Some studies suggest that immunogenicity is impaired in these patients, especially those treated with glucocorticoids, rituximab, mycophenolate mofetil, and abatacept.^28^ Since the vaccination rate of our patients during COVID-19 infection is very low, there is not enough evidence to assess the vaccine effect on patients’ outcomes.

Overall, almost 50% of the patients suspended one of their drugs in our study. There was no relationship between treatment modification and flare of disease activity. These findings support the recommendation of not discontinuing immunosuppressive drugs during the COVID-19 pandemic.^29^ The French Society of Rheumatology answers the most frequently asked questions during the pandemic, and it is recommended to continue baseline treatment if effective and well-tolerated to prevent exacerbations. If symptoms of COVID-19 infection are present, treatments should be withheld, and any corticosteroids should be maintained if prescribed.^30^

This study has some limitations. First, it is a retrospective single-centered study with a low number of patients which could limit more conclusive results. Second, there are some limitations to electronic health record-based databases, and some patients may remain undiagnosed. Those factors also could restrict our ability to assess disease activity. Third, a control group of non-IMIDs patients is lacking. However, this study compares patients with SpA and IBD with PCR-confirmed COVID-19, conducted with the active participation of the departments dealing with patients with IMID in our center.

In conclusion, this study shows that patients with IMIDs are not at increased risk of COVID-19 adverse outcomes despite the high use of biologics or immunosuppressants in this population. We also found that advanced age (>65 years), comorbidities, and bilateral ground-glass opacities on chest radiograph were associated with more severe infection requiring hospital admission. In addition, there were no significant differences in terms of COVID-19-related poor outcomes between SpA and IBD patients. Considering the autoimmune and autoinflammatory rheumatic diseases, much more data are necessary with large patient series to clearly understand the course and outcomes of COVID-19.

## Figures and Tables

**Table 1.** Baseline Characteristics of the Study Population

	SpA (n = 42)	IBD (n = 59)	Overall (n = 101)	*P*
Age, years, median (range)	48 (40-57)	46 (36-57)	47 (38-57)	.524
Male, n (%)	20 (47.6)	32 (54.2)	52 (51.5)	.512
Smoking habit, n (%)				
No smoker	33 (78.6)	44 (75.9)	77 (77.0)	.537
Past smoker	3 (7.1)	2 (3.4)	5 (5.0)	
Current smoker	6 (14.3)	12 (20.7)	18 (18.0)
Alcohol, n (%), none	41 (97.6)	57 (98.3)	98 (98.0)	.817
Educational level, n (%)				
Illiterate/primary education	16 (38.1)	21 (37.5)	37 (37.8)	.194
Lower/upper secondary education	16 (38.1)	16 (28.6)	32 (32.7)	
Post-secondary education	10 (23.8)	19 (33.9)	29 (29.6)
Type of occupation, n (%)				
Keep house	18 (42.9)	12 (21.4)	30 (30.6)	.265
Office employers	5 (11.9)	8 (14.3)	13 (13.3)	
Workers	7 (16.7)	13 (23.2)	20 (20.4)	
Retired or unemployed	7 (16.7)	10 (17.9)	17 (17.3)	
Other	5 (11.9)	13 (23.2)	18 (18.4)
Diagnosis, n (%)				
Psoriatic arthritis	17 (40.5)	-	17 (16.8)	
Ankylosing spondylitis	24 (57.1)	-	24 (23.8)	
Reactive arthritis	1 (2.4)	-	1 (1.0)	
Ulcerative colitis	-	47 (79.7)	47 (46.5)	
Crohn disease	-	11 (18.6)	11 (10.9)	
Unclassified	-	1 (1.7)	1 (1.0)
Disease duration (year), median (IQR)	7 (4-10)	8 (2-10)	7.5 (4-10)	.536
Active disease, n (%)	14(33)	10 (16.9)		.432
Comorbidities, n (%)				
None	25 (59.5)	33 (55.9)	58 (57.4)	.485
Diabetes mellitus	3 (7.1)	5 (8.5)	8 (7.9)	
Hypertension	3 (7.1)	2 (3.4)	5 (5.0)	
Cardiovascular disease	2 (4.8)	7 (11.9)	9 (8.9)	
Lung disease (e.g., asthma and COPD)	0 (0.0)	1 (1.7)	1 (1.0)	
Hypothyroidism	3 (7.1)	1 (1.7)	4 (4.0)	
Chronic liver disease	1 (2.4)	1 (1.7)	2 (2.0)	
FMF	2 (4.8)	0	2 (2.0)	
Psoriasis	0	2 (3.4)	2 (2.0)	
Rheumatoid arthritis	0	1 (1.7)	1 (1.0)	
Multiple sclerosis	0	1 (1.7)	1 (1.0)	
Baseline therapy, n (%)				
None	4 (9.5)	4 (6.8)	8 (7.9)	
NSAIDs	2 (4.8)	0 (0.0)	2 (2.0)	
CS, low dose alone or combo with MTX	3 (7.2)	2 (3.4)	5 (5.0)
**csDMARD **				
Sulfasalazine/mesalamine	6 (14.3)	39 (66.1)	45 (44.5)	
Colchicine	1 (2.4)	1 (1.7)	2 (2.0)	
Azathioprine	0	7 (11.9)	7 (7.0)	
Methotrexate	3 (7.1)	0	3 (3.0)
** ts/bDMARD **				
Anti-TNF alone or combo with 6MP/AZA/MTX/Leflunomide	19 (45.3)	5 (8.5)	24 (23.7)	
Jak inhibitor alone or combo with MTX	2 (4.8)	0	2 (2.0)	
Anti-integrin with CS high dose	0	1 (1.7)	1 (1.0)	
IL17 blockers with CS low dose+hidroksilorokin	2 (4.8)	0	2 (2.0)

AZA, azathioprine; CS, corticosteroids; COPD, chronic obstructive pulmonary disease; csDMARD, conventional synthetic disease-modifying antirheumatic drug; IL, interleukin; IQR, interquartile range; IBD, inflammatory bowel disease; MTX, methotrexate; NSAIDs, nonsteroidal anti-inflammatory drugs; SpA, spondyloarthropathies; TNF, tumor necrosis factor; ts/bDMARD, targeted synthetic or biological disease-modifying antirheumatic drug.

**Table 2.** Laboratory Findings After COVID-19 Diagnosis

Laboratory Values, Median (IQR)	SpA (n = 42)	IBD (n = 59)	Overall (n = 101)	*P*
Leukocytes, 1000/mL	5.96 (4.57-7.63)	6 (4.7-8.6)	6 (4.6-7.63)	.928
Lymphocytes, 1000/mL	1.92 (1.2-2.7)	1.6 (1.1-2.3)	1.79 (1.19-2.6)	.307
Hematocrit, %	39.8 (38-42)	39.9 (37.9-44.1)	39.8 (37.9-42.5)	.612
RBC, ×10^12^/L	4.5 (4.19-5)	4.7 (4.5-5.17)	4.61 (4.3-5)	.062
MPV, fL	7.9 (7.2-9)	7.4 (6.3-8.6)	7.84 (6.9-8.95)	.219
Creatinine, mg/dL	0.82 (0.62-0.91)	0.7 (0.6-0.9)	0.76 (0.6-0.9)	.340
Alanine aminotransferase, U/L	27 (21-43)	19.2 (14-24)	23.5 (15.5-32.5)	**.013**
Aspartate aminotransferase, U/L	27 (20-32)	23.5 (17-27)	24 (19-30.5)	.396
Total bilirubin, mg/dL	0.5 (0.32-0.62)	0.52 (0.4-0.83)	0.51 (0.37-0.7)	.213
C-reactive protein, mg/dL	25 (6.9-50)	15 (3-91.9)	17.75 (4.25-54.6)	1.000
Creatine kinase, IU/L	67.5 (45.5-108)	72.5 (54.91-89.7)	71 (50.6-105)	.929
Ferritin, ng/mL	152 (34-334)	73.4 (40.9-243.2)	128.5 (37.9-287)	.596
D-dimer, mg/mL	543 (229-1060)	382 (182-492)	425,5 (196-904)	.060
Fibrinogen, g/L	380 (340.5-470)	434 (426-483)	426 (373-483)	.462
Interleukin 6, pg/mL	-	107.9 (74.2-141.6)	107.9 (74.2-141.6)	NA
Troponin, ng/mL	0.6 (0.05-0.8)	1.2 (0.7-2.6)	1.05 (0.2-1.6)	.029

IBD, inflammatory bowel disease; MPV, mean platelet volume; SpA, spondyloarthropathies; RBC, red blood cell count; IQR, interquartile range; COVID-19, coronavirus disease 2019; NA, not available.

*P* < .05 is significant.

**Table 3.** Comparison of Baseline Clinical Characteristics and Outcomes of the Patients Infected with COVID-19 by Hospitalization

	SpA			IBD		
Hospitalization	Outpatient	*P*	Hospitalization	Outpatient	*P*
Overall, n	10	32		9	50	
Age, years	57 (46-66)	46.5 (37.5-53)	**.033**	60 (47-61)	43 (35-56)	.119
Age >65 years, n (%)	3 (30.0)	2 (6.3)	**.078**	2 (22.2)	4 (8)	.224
Male, n (%)	5 (50)	15 (46.9)	.863	6 (66.7)	26 (52)	.416
Smoking, n (%)						
Never smoker	9 (90)	24 (75)	.326	6 (75)	38 (76)	.286
Smoker	1 (10)	8 (25.1)	2 (25)	12 (24)
Disease duration, n (%)						
6 months-3 years	1 (10.0)	6 (18.8)	.805	2 (22.2)	12 (24)	.109
3-10 years	5 (50.0)	15 (46.9)		1 (11.1)	23 (46)	
>10 years	4 (40.0)	11 (34.4)	6 (66.7)	15 (30)
Comorbidities, n (%)						
0	6 (60)	19 (59.4)	.199	2 (22.2)	31 (62)	**.045**
1	2 (20)	12 (37.5)		5 (55.5)	16 (32)	
2	1 (10)	1 (3.1)		1 (11.1)	1 (2)	
3 or more	1 (10)	0	1 (11.1)	2 (4)
Biologics, n (%)						
Yes	4 (40.0)	19 (59.4)	.449	0	9 (18.0)	.269
No	6 (60.0)	13(40.6)	9 (100)	41 (82.0)	
Radiographic findings						
Bilateral interstitial abnormalities	1 (11.1)	1 (14.3)	**.016**	0	0	0
Ground-glass opacities (unilateral)	0	2 (28.6)		0	0	
Ground-glass opacities (bilateral)	**8 (88.9)**	**1 (14.3)**		7 (77.8)	5 (11.1)	
Normal findings	**0 **	**3 (42.9)**		2 (22.2)	3 (6.7)	
No imaging	0	0	0	37 (82.2)
Vaccination before COVID-19, n (%)						
None	10 (100)	30 (93.7)	.161	6 (66.6)	44 (88)	.638
Inactivated SARS-CoV-2 vaccine						
1 dose	0	0		1 (11.1)	2 (4)	
2 doses	0	1 (3.2)		2 (22.2)	4 (8)	
mRNA-based vaccine, 1 dose	0	1 (3.2)	0	0

IBD, inflammatory bowel disease; SpA, spondyloarthropathies; SARS-CoV-2, severe acute respiratory syndrome coronavirus-2; COVID-19, coronavirus disease 2019.

*P* < .05 is significant.
